# A Review of Dynamic Modeling Approaches and Their Application in Computational Strain Optimization for Metabolic Engineering

**DOI:** 10.3389/fmicb.2018.01690

**Published:** 2018-07-31

**Authors:** Osvaldo D. Kim, Miguel Rocha, Paulo Maia

**Affiliations:** ^1^SilicoLife Lda, Braga, Portugal; ^2^Centre of Biological Engineering, Universidade do Minho, Braga, Portugal

**Keywords:** dynamic modeling, strain optimization, phenotype prediction, metabolic engineering, hybrid modeling

## Abstract

Mathematical modeling is a key process to describe the behavior of biological networks. One of the most difficult challenges is to build models that allow quantitative predictions of the cells' states along time. Recently, this issue started to be tackled through novel *in silico* approaches, such as the reconstruction of dynamic models, the use of phenotype prediction methods, and pathway design via efficient strain optimization algorithms. The use of dynamic models, which include detailed kinetic information of the biological systems, potentially increases the scope of the applications and the accuracy of the phenotype predictions. New efforts in metabolic engineering aim at bridging the gap between this approach and other different paradigms of mathematical modeling, as constraint-based approaches. These strategies take advantage of the best features of each method, and deal with the most remarkable limitation—the lack of available experimental information—which affects the accuracy and feasibility of solutions. Parameter estimation helps to solve this problem, but adding more computational cost to the overall process. Moreover, the existing approaches include limitations such as their scalability, flexibility, convergence time of the simulations, among others. The aim is to establish a trade-off between the size of the model and the level of accuracy of the solutions. In this work, we review the state of the art of dynamic modeling and related methods used for metabolic engineering applications, including approaches based on hybrid modeling. We describe approaches developed to undertake issues regarding the mathematical formulation and the underlying optimization algorithms, and that address the phenotype prediction by including available kinetic rate laws of metabolic processes. Then, we discuss how these have been used and combined as the basis to build computational strain optimization methods for metabolic engineering purposes, how they lead to bi-level schemes that can be used in the industry, including a consideration of their limitations.

## Introduction

Systems biology and bioinformatics tools help to analyze relevant data and properties (e.g., genome sequencing) in biological and biomedical research to make model-driven discoveries. This has stimulated the interest to build genome-scale networks, allowing to perform *in silico* simulations of complex biological systems, and to understand the way metabolic flux distributions change within a particular biological network for predicting cellular phenotypes (McCloskey et al., 2013). Moreover, mathematical modeling of cellular metabolism, studied under various environmental and genetic conditions, has started to reasonably support metabolic engineering (ME) tasks, such as design of desirable strains, by optimal selection of gene deletions or expression modulation for the overproduction of industrial compounds (Stephanopoulos et al., 1998; Burgard et al., 2003).

Metabolic network modeling can be based on the knowledge of enzyme mechanisms and experimental data to build a representation of a dynamic system, able to describe changes on concentrations of metabolites over time by using systems of ordinary differential equations (ODEs). These ODEs contain initial values for metabolite concentrations, reaction rate equations and kinetic parameters. These representations were applied to model small-scale central metabolic pathways of well-known organisms, such as *Saccharomyces cerevisiae* (Rizzi et al., 1997) and *Escherichia coli* (Chassagnole et al., 2002). However, dynamic representations for large-scale systems are not always possible due to the lack of experimental kinetic information to build proper reaction rate equations.

This framework, that tackles cell metabolism modeling tasks using a formulation based on the dynamics of metabolic processes, gives detailed and unique solutions in time for the transient and the equilibrium states, from any initial metabolite concentration condition. It is based on kinetic rate laws inferred from biochemical and mechanistic information, while the values of the final fluxes are obtained directly from the rate laws and the metabolite concentrations at equilibrium. However, this type of modeling requires considerable amounts of data that are not always available, such as kinetic parameters or total enzyme concentrations (Smallbone et al., 2010, 2013), and the parameterization task for larger models can be time-consuming and computationally intensive.

Opposite to the dynamic case, an alternative is to restrict models to contain only reaction stoichiometry and reversibility, based on the assumption of steady-state operation, thus unable to express transient behaviors. In this approach, models use formulations based on linear equation systems, which are typically underdetermined, i.e., the number of equations is larger than the number of variables, translated into an infinite number of possible solutions. This leads to the imposition of certain restrictions (constraints) and assumptions (objective functions) to be able to find an optimal solution (Lewis et al., 2012). This formulation is based on the stoichiometry, via constraint-based modeling (CBM), helping to define limits on the behavior of a system depending on physical and chemical restrictions, such as fluxes, mass balance and thermodynamics. This approach yields solutions that might not be unique, represented as steady-state flux distributions, which are within the space of feasible solutions called the flux hypercone (Wagner and Urbanczik, 2005). While CBM approaches do not include physiological knowledge about metabolite concentrations in time nor transient behavior, they remove the need for a detailed mechanistic knowledge, since only parameters for minimum and maximum flux bounds are required. As a consequence, a solution space from a dynamic formulation is a subset of a constraint-based solution, since they use the same core constraints, knowing that the dynamic model adds other constraints from specific values of kinetic information (Machado et al., 2012).

**Figure 1:**
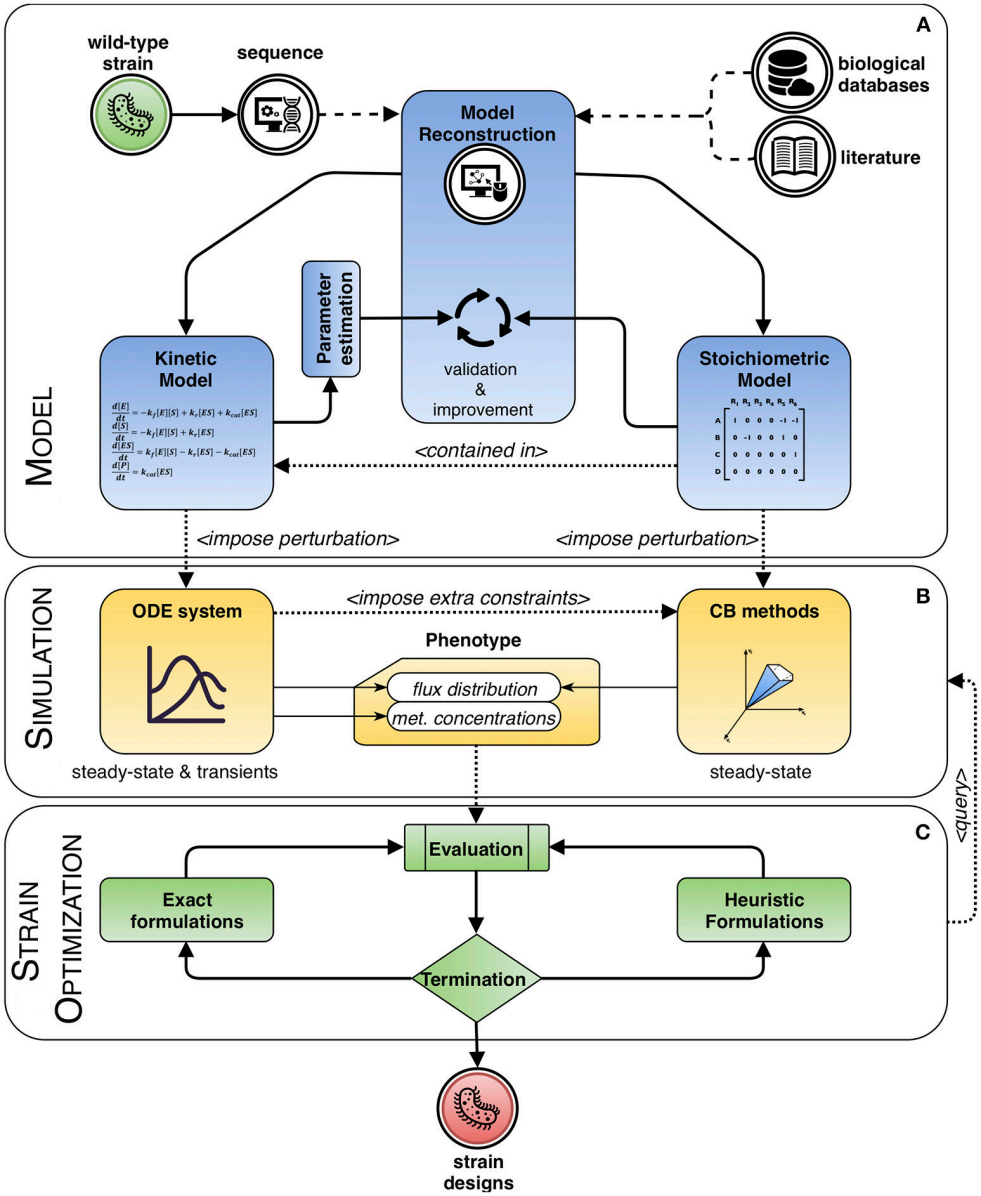
Modeling framework based on dynamic systems. The process of engineering a wild-type strain starts by reconstructing a model from its genome sequence complemented with information extracted from biological databases and literature. Then, the process can be divided in three main interacting blocks. **(A)** Model: it can include stoichiometric information only, or a combination with kinetics, whose parameters need to be estimated. Both types are validated and improved within an iterative process of curation. **(B)** Simulation: the model is used to predict the phenotype of the system. For kinetic approaches, the behavior of the steady and transient states of metabolite concentrations and fluxes are calculated, while with purely stoichiometry approaches, typically, a sensible flux distribution obeying the imposed constraints and optimizing a given biological assumption is sought. This flux distribution can be further delimited, in a hybrid fashion, by using information from the solution of the ordinary differential equation (ODE) system, if available. **(C)** Strain optimization: the phenotype is evaluated and optimized until meeting a termination criterion. The cycle consists in integrating solutions as perturbations to the model, in the form of changes to the kinetic parameters or to the constraints, so that a new phenotype can be simulated. In the end, a set of candidate designs is obtained.

Mathematical modeling can be used to explain or to predict the behavior of a system. This work is mainly focused on modeling frameworks based on the knowledge of systems' dynamics to increase the accuracy of the predictions of strain optimization methods, considering the limitations that the approaches can present. Computational strain optimization methods (CSOMs) depend both on mathematical models and phenotype prediction methods. We will review phenotype prediction methods that use kinetic rate laws for certain metabolites, and these will be used to support CSOMs for ME purposes. Additionally, hybrid approaches that include the use of kinetic and stoichiometric information will also be revised. Figure 1 describes the overall perspective of this review, which can be divided in three main parts: models, simulation (phenotype prediction) and strain optimization algorithms.

## Modeling formalisms and phenotype prediction

Mathematical modeling is used to understand internal and external cell interactions, and how they affect cell metabolism. This is studied through the analysis and modeling of phenotypes, as metabolite concentrations and reaction fluxes over metabolic pathways, regulated by enzymes under different internal and external conditions. The idea is to translate processes to numerical problems with formal representations, aiming a high level of accuracy and detail, since the goal is to reach enough complexity and completeness on the description of the behavior of a metabolic network.

The two types of approaches for mathematical representation of biological networks differ on the state at which the system is analyzed. Stationary modeling considers the system working at an equilibrium point, where metabolite concentrations are constant over time. On the other hand, dynamic modeling acknowledges the changes metabolite concentrations suffer over time. Both approaches for phenotype prediction will be discussed and compared in this section.

A straightforward approach for the prediction of metabolic phenotypes is using CBM, in the stationary case, which yields predictions usually close to experimental observations, and more importantly, avoiding the hard task of estimating kinetic rate equations and kinetic parameters, while requiring minimal knowledge to infer systemic properties. The most widely used mathematical approach to find an optimal value for the objective function and a relevant flux distribution is Flux Balance Analysis (FBA) (Orth et al., 2010). The main premise is to consider the internal metabolite concentrations to be in quasi-steady state under certain constraints, and formulating an optimization problem by means of the inclusion of an objective function with a biological meaning, for example assuming organisms evolve toward the maximization of their growth, which is usually modeled as a biomass formation artificial flux that includes proportions of cell constituents and their coefficients (Feist and Palsson, 2010). The assumption of microbes maximizing growth has been hard to assert, but it has been shown that under experimental conditions, such as nutritionally rich environments, there is a consistence between the optimization of growth rates and evolutionary principles (Varma and Palsson, 1994). However, under certain media/environment conditions or after genetic perturbations, the assumption might not be valid. Thus, other methods have been developed, namely: minimization of metabolic adjustment (MOMA), to test the hypothesis that knockout metabolic fluxes undergo a minimal redistribution with respect to the flux configuration of the wild type (Segrè et al., 2002); and regulatory on/off minimization (ROOM), to minimize the number of significant flux changes that have high growth rates with respect to the wild type (Shlomi et al., 2005).

In these CBM formulations, constraints are typically linear, such as capacity/reversibility constraints imposed by bounds on the values of the fluxes, and flux balance constraints imposed by stoichiometry. These constraints define an admissible solution space, with a general form of a polytope. Then, through the formulation of an optimization problem with a linear objective function, FBA identifies an optimal flux distribution by solving a linear programming problem.

Reviews and comparative analyses on available CBM methods have been performed, including procedures using different constraints, types of data and objective functions (Lewis et al., 2012), as well as studies on variants to address possible issues such as solution redundancy by internal cycles within networks (Smallbone and Simeonidis, 2009). However, besides the development of CBM methods, the need of having more detailed descriptions of biological networks has increased, leading to the addition of kinetic information.

### Dynamic models for phenotype prediction

Dynamic models (also known as kinetics-based models) to represent complex biochemical systems or processes have started to create a wide impact in the biotechnology industry for the design of novel cell factories (Wiechert and Noack, 2011). This is due to their potential in accurately predicting the effects of changing components in a metabolic network and to describe processes such as variation in metabolite concentrations and enzyme kinetics (Soh et al., 2012). The kinetics of a natural process, such as cell metabolism, along time are not usually linear or stationary; their representation inside a model uses mathematical expressions that describe the rates at which reactions are performed in the biochemical system in transient or stationary phases (Almquist et al., 2014). In fact, efforts to systematically construct dynamic models from genome-scale metabolic models have been developed recently, based on the use of data, such as reaction fluxes, metabolite concentrations and kinetic constants (Stanford et al., 2013).

In the context of industrial biotechnology, the goal for most cell factory models is linked to the comprehension and prediction of the effects of genetic or environmental perturbations applied to a system. Examples of these perturbations are the modulation of the expression of a metabolic enzyme, or changing the parameters of bioreactor processes, such as dilution rate or substrate uptake. Models built for specific applications, in particular for strain design, are focused in the production of industrial compounds, with an interest in maximizing the flux of the reaction related to the desired product among all outputs of the system. In the real world, the desired amount of a product can also be affected by cost, size of the model, initial substrates, nature of the process (metabolism, regulation and signaling) and environmental conditions (temperature, pH, type of bioreactor and timescale) (Demin and Goryanin, 2010).

The definition of the composition of a dynamic model entails the determination of the interaction mechanism subjacent to the network and the decision on the type of representation for the kinetic rate expressions. Then, the model can be described as a set of ODEs, commonly non-linear, which outlines the time trajectories of the represented processes and gets outputs that can be corroborated by experimental data for multiple time points, when they are available. In a deterministic and continuous context, ODEs combine stoichiometric information from the network organization, initial conditions and mathematical expressions for reaction rates as follows (DiStefano, 2013):

(1) $$\begin{array}{l} \frac{dx\left(t\right)}{dt}=S\cdot r\left(x\left(t\right),\;u\left(t\right),\;p\right)\\ y\left(t\right)=g\left(x\left(t\right),\;u\left(t\right),\;p\right)\\ with\;x\left(0\right)=x_{0}\left(p\right) \end{array}$$

where *S* is a matrix with dimension *i*×*j* that contains the stoichiometric coefficients for all the kinetic reactions in the network; *x(t)* is a vector of dimension *i* containing the time-dependent state variables; *r(*·*)* is a vector with dimension *j* including: reaction rates that depend on the state variables, a vector with input variables *u(t)* and a set of kinetic parameters *p*; and *g(*·*)* is a vector to relate model outputs *y*(*t*) with *x(t)*. For certain models, this representation is equipped with algebraic equations to complement the ODEs or with further differential equations that include volume changes, such as intracellular dilution for Equation (1) (Almquist et al., 2014).

One of the most used approaches to build a dynamic system is the forward or bottom-up modeling, which helps to integrate all the components and their interactions through mechanistic descriptions of the behavior, and characterizing the properties of the particular entities, from molecular to systemic levels, followed by experimental validation and refinement of the model. The components of kinetic models, seen as networks, are the rate expressions and parameter values that can be obtained from literature, experimental practices or data repositories. However, sometimes, parameters are unknown or undefined within certain ranges, which require the application of methods for parameter estimation, often a requirement of bottom-up approaches.

In contrast, top-down approaches, another way to address the reconstruction of dynamic models, use experimental data to improve pre-existing models that predict measured data successfully. In this way, unidentified mechanisms, interactions and properties can be integrated into the model. The trend will always be to formulate objectives using models able to capture high-level mechanisms with simpler rate expressions, taking into account essential links between network components (Bruggeman and Westerhoff, 2007).

In the next subsections, we will examine two key points to build dynamic models for phenotype prediction: the network structure, including interactions, and the mathematical expressions to represent the change in reactions. Afterwards, the possible techniques to study kinetic parameters are studied.

#### Interaction network

The internal and external components of the model communicate through a network that can be represented using a diagram, as a group of links between metabolites, enzymes, reactions, among others. This representation collects the required and available biological information to be converted into a mathematical system, allowing functionality to be derived from the structure. Biological systems are usually seen as networks, since different properties can be analyzed as an engineered system, such as robustness, closed-loops and modularity (Villaverde and Banga, 2014). However, dynamic behavior, stoichiometry and parameters are not accounted for in interaction-based approaches, for which the use of different mathematical tools is introduced to improve their description, according to the type of analysis. An overview of the key computational and statistical concepts and methods to reconstruct cellular networks has been provided by Markowetz and Spang (2007).

#### Kinetic rate expressions

Mathematical expressions can describe behavior of interactions as rates of change. Their complexity depends on the degree of detail of the reactions and on the scope of the model, taking into account that future model reductions can be performed after finding a balance between the model and the validation data. Kinetic rates are representations derived from deterministic or stochastic formulations. However, this distinction can be arbitrary, since all types of kinetic expressions are approximations, as will be described next. In the deterministic case, there are mechanistic expressions from physico-chemical reaction processes, or approximate kinetic expressions that are mostly qualitative (Demin and Goryanin, 2010).

##### Mechanistic expressions

The primary kind of mechanistic reaction kinetics is based on the mass action law, stating that a reaction rate is proportional to the concentrations of the reactants. This representation is used for one-step or elementary reactions, or the combination of their mass actions for multi-step reactions, e.g., for enzymatic or transporter reactions.

*Michaelis-menten*. The basic mechanistic expressions are the Michaelis-Menten kinetics (Bertolazzi, 2005), that are derived from one-step reactions by splitting fast and slow dynamics. They are commonly used for cases where the enzyme concentration is much lower than the substrate concentration, using the following form to describe the rate of the enzymatic reaction:

(2) $$\begin{array}{l} r=\frac{d\left[X\right]}{dt}=r_{\max}\frac{\left[S\right]}{\left[S\right]+k_{m}} \end{array}$$

where *r* is the reaction rate, *[S]* is the concentration of the substrate, *[X]* is the concentration of the product, *r*_*max*_ represents the maximum rate reached by the system, and the Michaelis constant *k*_*m*_ represents the substrate concentration at which the reaction rate is at half-maximum (*r* = *0.5r*_*max*_). The mechanisms in this formulation are often very complex and not well-known, and can include nutrients and inhibitors as limiting substances; these considerations and analytical derivation of laws are detailed in Bertolazzi (2005). For this reason, the determination of kinetic rate expressions is complex and processes have to be carefully studied by experimental means under the same conditions assumed by the model. In these cases, there are a large number of parameters to determine, usually a set of maximum rates and kinetic constants for each reaction. In addition, the level of uncertainty has to be analyzed, because predicted fluxes and metabolite concentrations should be within acceptable ranges. The uncertainty of mathematical models represents limitations in applicability and in completeness, as well as in the effect of other factors, such as temperature, pH, ionic strength, among others (Almquist et al., 2014).

*Hill rate laws*. Hill rate laws are used as a modeling approximation for transfer functions that involve regulation within a biological system (DiStefano, 2013). A Hill function of order *n* with associated parameters *p* = *{n, k*, λ*}* can represent activity as follows:

(3) $$\begin{array}{l} \frac{dx_{act}\left(t\right)}{dt}=f_{act}\left[x\left(t,p\right)\right]=\lambda \frac{x^{n}}{x^{n}+k^{n}} \end{array}$$

(4) $$\begin{array}{l} \frac{dx_{inh}\left(t\right)}{dt}=f_{inh}\left[x\left(t,p\right)\right]=\lambda \frac{k^{n}}{x^{n}+k^{n}} \end{array}$$

where Equation (3) represents an activation, Equation (4) represents an inhibition, *x*_*act*_ and *x*_*inh*_ are (respectively) the activated and inhibited substrates (depending on time and certain parameters), λ is the maximum regulation rate, *k* is the activation or inhibition coefficient, and *x* is the substrate concentration. It is noticeable that if *n* = *1*, the Hill function for activation is the same as the Michaelis-Menten equation (known also as Monod equation).

##### Approximate kinetic expressions

In comparison with mechanistic expressions, there are some alternatives to describe the behavior of biological systems in a simpler way, using analytic functions as power series that always converge avoiding unstable systems. Also, a small amount of parameters needs to be determined, since most rate expressions are not known or are difficult to quantify, but a universal formula can be applied to the reaction kinetics (Borger et al., 2007). This is translated into less efforts to estimate parameter values, added to a possible few experimental data available. These approximate formulations were compared with mechanistic approaches for some modeling cases, resulting in similar model behavior with respect to transient and steady states, even with the use of general formulations and less parameters. Additionally, hybrid approaches have been studied to combine mechanistic and approximate kinetics having also suitable results (Bulik et al., 2009). Relevant approximate rate expressions for dynamic modeling are described next.

*Lin-log kinetics*. Lin-log kinetics is a linear representation of logarithms for large concentrations of species, such as enzyme mechanisms, with the rate of catalyzed enzyme reaction proportional to its concentration and dependent on parameters and metabolite concentrations (Heijnen, 2005). These are a factor of the linear sum and are able to provide analytic solutions for the steady states (del Rosario et al., 2008). The Lin-log rate law *r* of the *j*^th^ enzyme catalyzed reaction, *N*, in a network with *M* species, is expressed as:

(5) $$\begin{array}{l} r_{j}=\frac{e_{j}}{e_{j}^{0}}J_{j}^{0}\left(1+\overset{M}{\underset{k=1}{\sum }}\varepsilon_{j,k}^{0}\log\frac{N_{k}}{N_{k}^{0}}\right) \end{array}$$

where superscripts denote steady-state, *e/e*^0^ is the enzyme activity relative to the reference steady-state (*e*^0^), *J* is the flux for the corresponding reaction, ε is the elasticity coefficient (EC) that measures sensitivities of metabolite concentrations on the reaction rate, and *N/N*^0^ is the relative concentration of metabolites for metabolite *k*.

*Log-lin kinetics*. Similar to lin-log, this formulation allows to get analytic solutions through linear and logarithmic expressions on reactants and effectors to approximate reaction rates (Hatzimanikatis and Bailey, 1997). In this case, the reaction rate is not proportional to the enzyme concentrations since the last ones are inside the linear terms.

*Power-laws*. Here, substrates and products are related to approximate the reactions rates as:

(6) $$\begin{array}{l} r=\lambda \overset{n_{subs}}{\underset{j=1}{\prod }}{\left(\frac{S_{j}}{S_{j}^{0}}\right)}^{m_{j}}-\mu \overset{n_{prod}}{\underset{k=1}{\prod }}{\left(\frac{P_{k}}{P_{k}^{0}}\right)}^{n_{k}} \end{array}$$

where λ and μ are the aggregations of the forward and reverse rates that interact with substrate *S* and product *P*, respectively, superscripts denote a reference state, *m* and *n* are the kinetic orders of the respective compound, corresponding to the exponents in the reaction equation by which each concentration term is powered to, while *n*_*subs*_ and *n*_*prod*_ are the number of substrates and products, respectively.

There are two main types of forms for power-law kinetics (Savageau, 1970): 1) Generalized mass-action, describing reactions with non-integer exponents and rates unfolded for each reaction that interacts with the species (similar to mass-action formulation), allowing at the same time an analytic solution at steady-state; 2) S-systems, for forward and reverse kinetics, where individual reaction rates aggregate two reactions for every mass balanced species, capturing non-linearities at the local state, and giving analytic solutions of steady-states.

*Convenience rate laws*. These approximations are derived from an enzyme mechanism without order, assuming a fast equilibrium between substrates, products and enzymes. The formulation allows concentrations close to zero, which can bring problems with logarithmic representations. The rate laws assume an enzyme mechanism of random order and can be applied to reactions with any number of substrates and products. This is a more general form of Michaelis–Menten kinetics that involves extensive reaction stoichiometries (Liebermeister and Klipp, 2006).

*Modular rate laws*. These expressions are approximate kinetics that involves the representation of a family of semi-mechanistic approaches (Liebermeister et al., 2010) in the standard form:

(7) $$\begin{array}{l} r=T\frac{E_{0}\cdot f_{r}}{D+D^{reg}} \end{array}$$

where *r* is the modular rate law, *T* is a stoichiometric parameterization term, *E*_0_ is the initial concentration of the enzyme, *f*_*r*_ is a complete or partial regulation, *D* is a denominator term for every rate law and *D*^*reg*^ is the particular regulation term.

*Cooperativity and saturation*. Biochemical networks and their processes can be modeled with cooperativity and saturation by using a canonical formalism including equations similar to Hill rate laws. They include a local representation given an operating point, based on a functional form derived from Taylor series approximations in a special transformation space, defined by power-inverses and logarithms of power-inverses (Sorribas et al., 2007). Moreover, the formalism can be used as an extension of power laws with a bigger accuracy for numerical simulations, and to explore predicted solutions coming from constraints of local sensitivities and different saturation fractions.

##### Stochastic kinetic expressions

Deterministic formulations of reaction kinetics are realistic when the number of reacting molecules is large per reactant, which is the case of the most common modeled cell factories. However, for small numbers of chemical species in relevant applications, stochastic behavior may happen, such as in signaling or gene expression, for which stochastic simulation approaches are taken into account. The most common formulation for stochastic models is the chemical master equation (Ullah and Wolkenhauer, 2010). This approach introduces new insights to the field since solutions can satisfy conditions that change in time. Deviations, in the form of noise, are included in a chemically reacting system, which can explain connections between stochastic equations and deterministic rate laws. This kind of biochemical networks can exist in continuous or discrete state spaces as explained next.

For continuous spaces, stochastic simulations use analytic approximations for the influence of randomness on the behavior of a system. The representation is through stochastic differential (or Langevin) equations, which can be derived from corresponding deterministic partial differential equations for the kinetics of the probability distribution of the molecules (Gillespie, 2000).

In the case of discrete spaces, the basic idea is that the state of the system comes from the exact numbers of molecules, where the changes of reaction states are described by the probabilities of the transitions between every possible state (property known as reaction propensity). The formulation also includes a master differential equation that holds the time evolution of the state probabilities, which can be described by a non-trivial stochastic simulation algorithm (Gillespie, 1976).

#### Discussion

To summarize approaches for phenotype prediction, Table 1 provides an overview of the dynamic modeling methods described in this review. It provides information about the reasons for using the different algorithms, their advantages and disadvantages, and proposes illustrative examples of their application. In the three following paragraphs, one example from each class of methods—mechanistic, approximate and stochastic—is detailed showing how each modeling approach helped to better understand metabolic pathways for phenotype prediction.

**Table 1:** Classification of kinetic rate expressions.

**Type**	**Rate law**	**When to use it?**	**Advantages (+)/Disadvantages (–)**	**Example**	**References**
Mechanistic	Michaelis- Menten	To have the basic mechanistic expressions. With kinetics where enzyme concentration is much lower than the substrate concentration. To model the summation of the effects of two or more reversible inhibitors or activators	(+) For very complex and not fully-understood mechanisms. (+) Knowledge of kinetic constants for substrates or regulators is not required since they can be estimated	Pharmacokinetic model	Sheiner and Beal, 1980
	Hill rate laws	To model structure functional features of molecular genetic systems that do not demand knowledge of their detailed mechanism	(+) Suitable for not well-known molecular mechanisms by using generalized functions	Regulation of the expression of the *cydAB* operon in *E. coli*	Likhoshvai and Ratushny, 2007
Approximate	Lin-log	In gene regulatory systems where rates are proportional to enzyme levels. With number of parameters as small as possible	(+) Analytic solution of steady-state network balances are desirable. (-) Parameter estimation methods not always fit satisfactory the data, especially when having small concentrations.	Glycolisis in *Lactococcus lactis*	del Rosario et al., 2008
	Log-lin	For metabolic systems subject to spatiotemporal variations of system parameters and the process operating conditions.	(+) Accurately describes dynamic responses of strongly non-linear systems with analytic solutions. (+) Based on metabolic control analysis data to study parameters. (–) Quasi-steady-state approximation.	Yeast glycolytic system	Hatzimanikatis and Bailey, 1997
	Power laws	For arbitrary systems of enzyme-catalyzed reactions. Able to simply model aggregation and consumption processes	(+) Suitable solution approximation for enormous non-linear chemical systems using conventional numerical methods	Basic growth of complex systems	Savageau, 1979
	Convenience rate laws	To represent enzyme saturation and regulation by activators and inhibitors. It uses thermodynamically independent system parameters	(+) Small number of parameters that can be easily computed with least-squares estimation methods	Chinese hamster ovary cell metabolism	Nolan and Lee, 2011
	Modular rate laws	In reversible rate laws for reactions with arbitrary stoichiometries and various types of regulation	(+) Simplifies thermodynamic-kinetic modeling formalisms being flexible and biochemically plausible. (−) Less accurate than detailed kinetic equations	Cycle of three reactions (illustrative example)	Liebermeister et al., 2010
	Cooperativity and saturation	To fit experimental data using systems with saturable form	(+) Expected to be accurate over a wider range around the operating point if the approximated functions are saturated. (−) Need of a large number of parameters (increased estimation efforts). (−) Common canonical formalisms do not have saturable form	Illustrative example of metabolic network with one positive feedforward and one negative feedback	Sorribas et al., 2007
Stochastic	Continuous space	For stiff systems, which can evolve on slow and fast time scales, and having stability in the fastest modes	(+) Able to describe the common enzyme-catalyzed conversion of a substrate into a product. Dramatically speeds up the stiff reactions.	Simulation of the general stiff enzyme-substrate reaction	Cao et al., 2005
	Discrete space	For problems with identifiability issues, when changes in the species are discrete and random, rather than continuous and deterministic	(+) Capture variability for bistable systems; able to deal with noise (randomness)	Isomerization of proteins	Ullah and Wolkenhauer, 2010

*A summary of dynamic modeling methods, condensing information about the cases for using the different methods, their advantages and disadvantages, and an illustrative example of their application*.

Mechanistic modeling methods do not demand knowledge of the detailed mechanisms of a system by using conventional expressions to describe structural features of metabolic systems, as well as to model the added effect of two or more reversible inhibitors or activators. This approach is combined with the fact of kinetic parameters able to be estimated with traditional methods since they are not always available. One notable example is the generalized Hill function that can be used when molecular mechanisms are not well understood. The methods consider experimental data regarding structural/functional features of a system, and descriptive data of its dynamics. For instance, available data was used to describe the expression regulation of the *cydAB* operon in *E. coli* to further understand metabolic activity of its cells under different conditions (Likhoshvai and Ratushny, 2007). The method describes the changes of transcription factor concentrations that affect the rate of enzymatic reactions, depending on oxygen concentrations. The result is a model predicting the level of *cydAB* expression in agreement with available experimental data and simulation results. The use of generalized Hill functions allowed to bypass the problems of reconstructing the detailed mechanisms of the molecular subsystems.

Approximate modeling methods are typically used to facilitate the analysis and design of strongly non-linear pathways, using simpler universal expressions in the form of analytical functions. One example of their use is the log-linear approach, used together with available data on elasticities and control coefficients, to understand glycolytic pathways in yeast, which present a strong non-linear behavior (Hatzimanikatis and Bailey, 1997). The analytical solution of the log-linear model for a number of metabolites and enzymatically catalyzed reactions, depends explicitly on information from metabolic control analysis (MCA) (Fell, 1992). This solution considers a linearization around a steady-state using logarithmic deviations of the state variables and parameters. Studies can be performed regarding the effect of modifications of the catalytic properties of an enzyme with respect to its substrate (or regulatory effector), by changing the value of the corresponding elasticity. Time-response of fluxes show an excellent agreement between the original non-linear model and the log-linear model (Hatzimanikatis and Bailey, 1997). However, the average performance has limitations under quasi steady-state, for which the prediction of metabolic functions can be deteriorated.

Stochastic modeling methods are commonly applied for systems with small number of chemical species, to describe processes that present deviations, e.g., noise in gene expressions or signaling, giving solutions that can satisfy conditions that change in time. Moreover, applied as a non-deterministic approach in continuous spaces, these methods can be used to describe common stiff reaction motifs in cellular metabolic systems, for instance, the enzyme-catalyzed conversion of a substrate into a product or its decay into its original constituents. The reactions can be divided in fast and slow time scales, and the simulations can reach very accurate levels under certain conditions (Cao et al., 2005). The time trajectories of the species of the model are simulated using random sampling, an approach that results in satisfying the formulation given by the deterministic Michaelis-Menten derivation. Thus, this stochastic method is useful when a difference of speed can be found in the stages of the reaction, with the advantage of having simulations dramatically faster without noticeable loss of computational accuracy.

**Figure 2:**
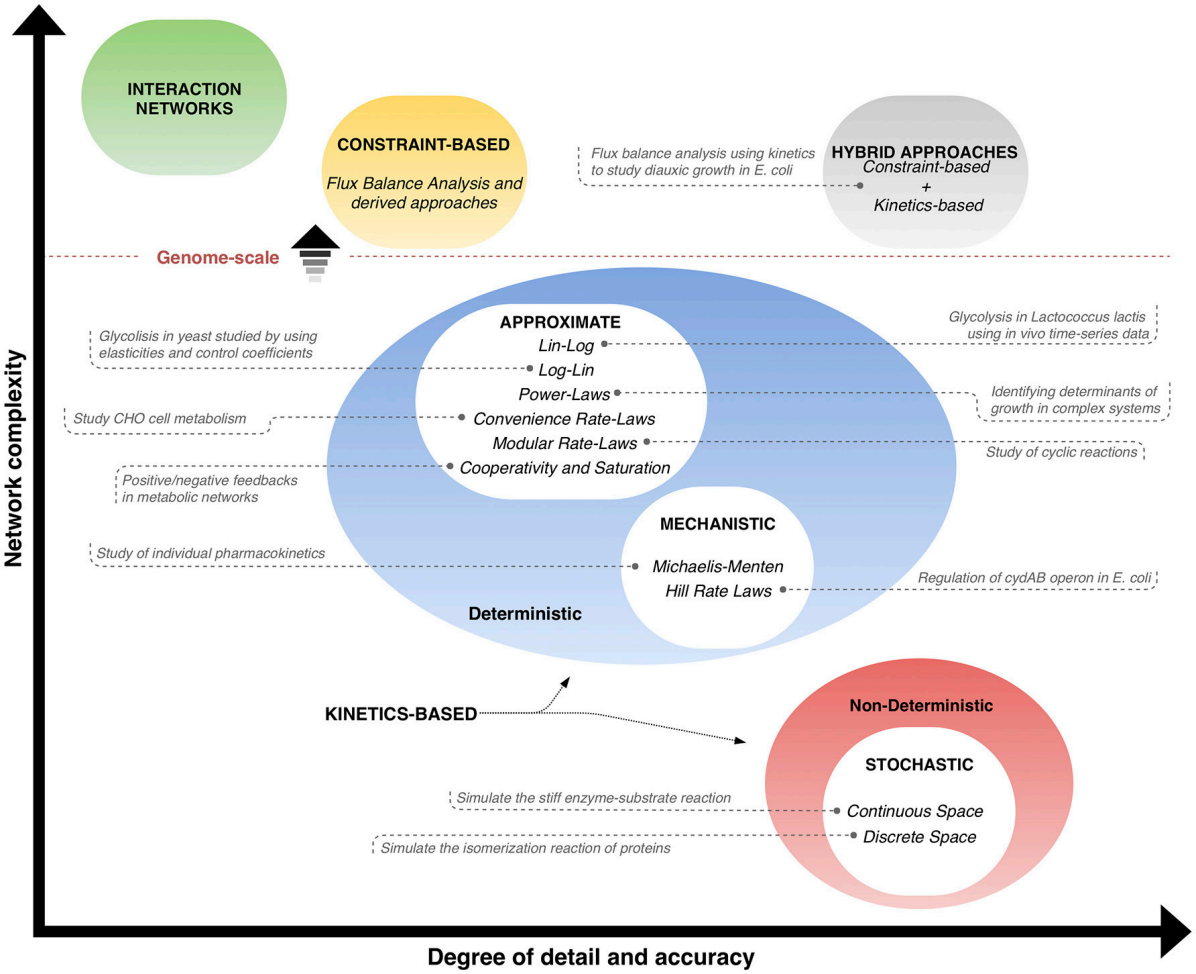
Phenotype prediction approaches: interaction networks, constraint-based and kinetics-based methods can be classified qualitatively according to the level of detail and accuracy (horizontal axis), and the usual size of network (vertical axis). Position toward upper levels means genome-scale networks. Constraint-based and kinetics-based approaches can be joined in hybrid methods that aim at taking the best advantages of each of them. Examples of applications of kinetics-based and hybrid approaches are given (from Tables 1, 3, respectively).

The different classes of kinetics-based methods used for phenotype prediction (explained in the previous subsections) are illustrated in Figure 2, and a qualitative comparison is made with respect to interaction networks, constraint-based methods and hybrid approaches. The classification takes into account the complexity in size (vertical axis) and the level of detail/accuracy (horizontal axis) to describe the systems, and places each method within these coordinates, where positions toward upper levels mean genome-scale networks. Interaction networks are placed at the top in terms of network complexity, but contain few information and details of the behavior of the entities. Similarly, constraint-based models not only consider a big amount of interactions, but also provide more information about the properties of reaction rates happening; however, the detail on how the behavior of the reactions in time is low. On the other hand, kinetics-based models are split in the graph space according to their deterministic or non-deterministic nature. Deterministic approaches include approximate and mechanistic methods, which have in average a medium level of complexity and degree of detail/accuracy, since they take into account dynamic information. Non-deterministic methods refer to stochastic approaches, which can describe the operation of the systems with high detail and accuracy, but are typically limited in network size. Finally, hybrid approaches, a combination of stoichiometric and dynamic information, are positioned with a high level of accuracy on the description of high-sized networks, since the best features of two approaches are integrated into a single one. The trend of research is to find methods that can predict behavior of bigger networks with more detail and accuracy. Examples of applications of kinetics-based and hybrid approaches are shown in the graph. Interaction networks and constraint-based approaches are only included in the qualitative comparisons and are not considered further, since they are out of the scope of this review and have been thoroughly examined in other reviews (Markowetz and Spang, 2007; Lewis et al., 2012).

### Parameterization techniques

The measurements obtained from experiments help to determine the values of individual parameters for kinetic rate expressions, initial conditions and outputs. These values can be found in data repositories that compile this information such as BRENDA, which gives a collection of enzyme and metabolic information (Schomburg et al., 2004), and SABIO-RK, that stores information about biochemical reactions and their kinetic properties (Wittig et al., 2012). In case measurements are not available, parameter values can be estimated by inference, using existing experimental data, and estimation methods that can be constrained with thermodynamic and physical/chemical conditions to assure that values are unique (Chakrabarti et al., 2013).

Kinetic parameters are found either all simultaneously, by making the model fit the measurements of the whole system, or one by one considering individual components and processes. Furthermore, both approaches are usually combined by fixing parameters to already known values and fitting the remaining ones. However, different parameter values are often found from different sources, in distinct experimental conditions, which brings compatibility problems. In addition to the parameter estimation, a study can be done on how changes on parameters affect the behavior of a model. Both methods will be reviewed in the next subsections.

#### Parameter estimation

To avoid compatibility problems from finding parameters from different sources, parameter estimation techniques are used, indirect methods allowing optimal values of parameters to be calibrated as a solution to an estimation problem, making the model reproduce experimental measurements of different values instead of the parameter themselves. Moreover, there are numerical optimization algorithms, with stochastic or deterministic approaches, that allow to determine the quality of experimental data in an efficient and automated way, making the data generated by different measurement methods reliable for quantitative dynamic modeling (Raue et al., 2013). The critical consequences of the limited availability of kinetic data in metabolic dynamic modeling have been discussed with respect to specific organisms. The study concludes a remarkable necessity for producing curated data to approximate *in vitro* conditions to the *in vivo* ones, so that an integration of available kinetic data into a complete large scale model is possible (Costa et al., 2011).

Parameter estimation follows an optimization algorithm that searches through a large set of possible values, under certain constraints and non-linear structures, which can imply complex objective functions with multiple solutions in the form of local optima. The goal of optimization algorithms is to locate a global optimal in a feasible time, using local or global methods.

Local methods have to initiate the optimization with reference parameters that can be measured experimentally or found in literature, and then improved after repeating the execution of the algorithm. The algorithms are commonly based on the Hessian and gradient of an objective function, usually computed by numerical methods, such as finite difference approximations. However, this can bring problems with speed of convergence for complex structures. In addition, local methods find optimal solutions in some feasible neighborhood that are not always the global solution, unless the region of feasible solutions is convex (Nocedal and Wright, 2006).

On the other hand, global methods are based on metaheuristics, such as simulated annealing (Kirkpatrick et al., 1983), genetic or evolutionary algorithms (EAs) (Sarkar and Modak, 2003; Yüzgeç et al., 2009). The combination of global and local methods has been the most successful tool to explore the parameter space when solutions are close to an optimal (Moles et al., 2003). The objective of metaheuristic methods used for the parameter estimation task is to accelerate the process for large-scale systems biology models (that are usually non-linear dynamic systems). This can be achieved with parallel and self-adaptive cooperative strategies based on scatter search optimization, which can significantly reduce computation times, and improve performance and robustness (Penas et al., 2017).

The classical approach for the objective function, when performing the estimation of parameters, consists in minimizing the difference between the model output and the experimental data (Chou and Voit, 2009). The formulation considers model outputs in vector *y(*·*), M* data points in a vector *p* = *(p*_1_…*p*_*M*_*)* measured at times *(t*_1_…*t*_*M*_*)*, θ as the set of parameters to be estimated, and an objective function Φ*(*θ*)* defined by the distance of the vector residuals [*p*_1_ *- y(t*_1_, θ*), …, p*_*M*_ *- y(t*_*M*_, θ*)*], that usually is seen as the weighted sum of squares. The formulation of the objective function is described as follows:

(8) $$\begin{array}{l} \Phi \left(\theta \right)=\overset{M}{\underset{j=1}{\sum }}\frac{{\left(p_{j}-y\left(t_{j},\theta \right)\right)}^{2}}{\sigma_{j}^{2}} \end{array}$$

where $\sigma_{j}^{2}$ is the error weight for the *j-*th data point. Then, the values to be estimated, θ^*^ are the ones that minimize Φ*(*θ*)* (Raue et al., 2009).

Furthermore, the parameter estimation can be seen as a geometrical problem, as stated previously, or also as a statistical formulation (Ljung. L., 1987) that takes the experimental data as events of random variables. The model deviation on the prediction is defined as ε_*j*_ and added to the output for each data point as *p*_*j*_ = *y(t*_*j*_*)* + ε_*j*_, with which the observed output in Eq. (1) is modified to *y(t)* = *g(x(t), u(t)*, θ*)* + ε. Assuming independent and normally distributed deviations, the likelihood Λ of observed data points *p* with the rest of variables defined in Equation (8) is:

(9) $$\begin{array}{l} \Lambda \left(\theta \right)=k\overset{M}{\underset{j=1}{\prod }}\exp\left[-\frac{{\left(p_{j}-y\left(t_{j},\theta \right)\right)}^{2}}{2\sigma_{j}^{2}}\right] \end{array}$$

where *k* is a constant that does not affect the optimal likelihood. Additionally, θ^*^ is defined as the maximum likelihood estimate for θ that optimizes Λ*(*θ*)*, which leads to rewrite the problem as the minimization of the negative logarithm of the likelihood function, that is equivalent to the geometrical formulation. Likelihood theory is equivalent to least squares theory, and it yields identical estimators of the structural parameters (except for the variance) for linear and nonlinear models when the error terms are assumed to be independent and normally distributed (Burnham and Anderson, 2002)

Other ways to find optimal values for parameters include using adjoint sensitivity analysis for the purpose of accurate gradient estimation, with a superior scalability compared to the standard forward sensitivity-based optimization, which has a level of complexity of systems independent of the number of parameters to estimate (Fröhlich et al., 2017a). Additionally, optimal parameters can be found by exploiting the local geometry of the steady-state manifold and its stability properties, due to the dynamics of the process restricted by steady-state constraints, such as initial conditions at equilibrium (Fiedler et al., 2016). Moreover, optimization of parameters can be achieved by describing the sensitivity equations for a gradient computation problem that includes event-resolved data using event triggered observations (Fröhlich et al., 2017b). Further, there are computational optimization tools available that implement metaheuristic methods for parameter estimation that can be applied to multiple domains of systems biology and bioinformatics, such as the MEIGO toolbox (Egea et al., 2014). Finally, global optimization for non-linear dynamic models has been presented as a solution for improving computation times in comparison with deterministic global methods (Rodriguez-Fernandez et al., 2006).

An important procedure performed in parallel to parameter estimation is to study the uniqueness and level of confidence of the variables that are going to be computed. For that, performing identifiability analysis, local or global, is essential to evaluate the goodness of experimental data to determine model parameters. However, certain models are not identifiable according to their structure, based on known inputs and measured outputs, which turns parameter estimation meaningless. Structural identifiability analysis helps to know which quantities have to be measured and which are able to be estimated. Theory and tools available for the study of identifiability have been previously reviewed and discussed, together with related concepts such as sensitivity to parameter perturbations, observability, distinguishability, and optimal experimental design (Villaverde and Barreiro, 2016).

Many algorithms have been developed for this task, one in particular using observability, to know how the internal states of a rational model can be inferred by the nature of its outputs. This method is based on computing the rank of a numerically instantiated Jacobian matrix (observability/identifiability matrix) to evaluate the local structural identifiability (Sedoglavic, 2002; Karlsson et al., 2012). Moreover, computational tools have been exploited to analyze the structural identifiability of a very general class of nonlinear models by extending previous methods, and also showing how to modify unidentifiable models to make them identifiable (Villaverde et al., 2016a). Besides these tools, methods to analyze global structural identifiability for arbitrary model parameterization have been developed (Ljung and Glad, 1994), as well as to assess local structural identifiability for a general non-linear state-space model (Stigter and Molenaar, 2015).

Furthermore, to evaluate the accuracy of estimated parameters, it is common to analyze standard parameter confidence intervals, defined as a quadratic approximation of the logarithmic likelihood around the optimal value (Raue et al., 2011) or, alternatively, calculating exact confidence intervals from a threshold level in the likelihood, where parameter directions are explored, while likelihood is minimized with respect to other parameters (Raue et al., 2009).

**Figure 3:**
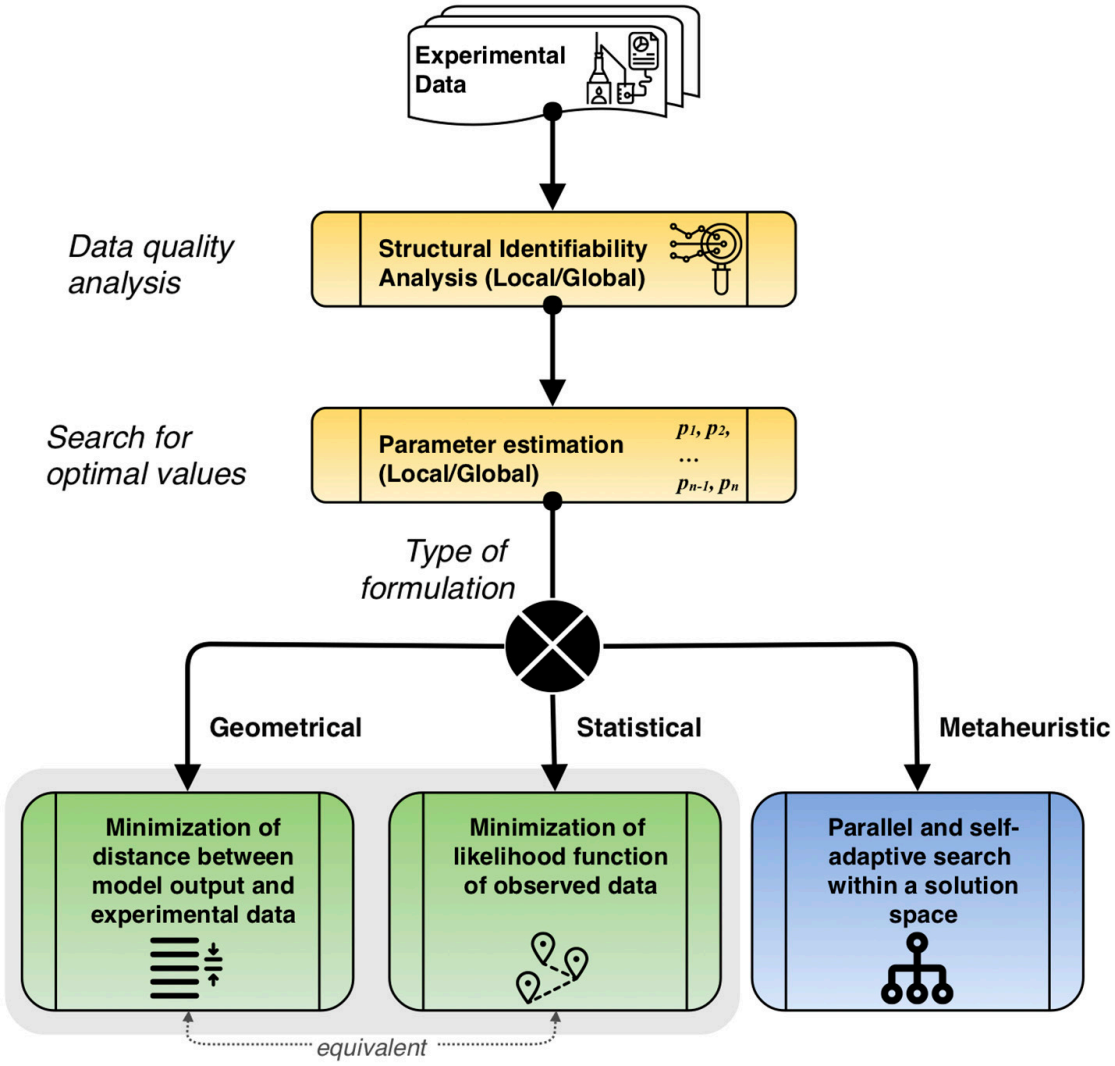
Description of the overall parameter estimation procedure. First, quality of experimental data is studied to determine suitable parameters via structural identifiability analysis. Then, parameter estimation is performed, locally or globally, according to the type of problem formulation. The equivalence between the geometrical and statistical formulations is noted.

The general parameter estimation procedure is described in Figure 3, considering the main types of approaches. Before estimating parameters, a study of the dynamic data has to be performed using structural identifiability. This process helps to qualitatively assess if the available data is useful to make suitable predictions. After this, the search for optimal values that follows depends on the type of approach chosen: geometrical, minimizing the distance between the model output and experimental data, which is equivalent to the statistical formulation that minimizes the likelihood function of observed data, or metaheuristic, that performs a parallel and self-adaptive search within a solution space.

#### Local parameter sensitivity analysis

This type of study allows to identify how a model varies its behavior, such as changes in fluxes and metabolite concentrations, in response to a perturbation around some points in the parameter space. This analysis can be done through genetic modifications affecting enzyme concentrations, which will allow to identify reasonable ME targets that affect positively the behavior of a cell factory. This kind of sensitivity analysis for dynamic models can be performed through methods such as MCA (Fell, 1992). MCA quantifies, through two different dimensionless indices, how the control of a flux in equilibrium state is distributed among the enzyme reactions in a particular pathway, namely elasticity coefficients (ECs) and flux control coefficients (FCCs). ECs are defined using metabolite concentrations and reaction rates catalyzed by enzymes with particular concentrations. FCCs for any flux in steady-state show the degree of control of enzymes on the pathway of that specific flux.

ECs and FCCs help to connect properties of the system and components, using the fact that for each metabolite *k*, the sum of the product of ECs and FCCs is zero with respect to that metabolite. Larger values of FCCs indicate that the corresponding reactions are primarily controlling the flux, leading to target those enzymes for a successful ME of the corresponding pathways; details on MCA method are further discussed in Fell (1992). Moreover, MCA can be applied to steady-state fluxes and metabolite concentrations, and combined with parameter sampling approaches to analyze parameter uncertainties (Wang et al., 2004). Some extensions of MCA have been developed for more significant modifications (Nikolaev, 2010), since a model with good predictive power is required to simulate larger changes in structure or parameters of the model, which means robustness with respect to different operating references. For instance, candidate targets, after MCA increases the production of some compound, can suggest large changes in two or more enzyme concentrations by firstly simulating a deletion strategy. Then, by overexpressing an enzyme and analyzing how pathways were affected, this results in a combination of two modifications that can improve a certain flux in the desired direction, compared to the results of the wild-type strain (Hoefnagel et al., 2002).

### Efforts on modeling the metabolism of *E. coli*

The development of dynamic models to quantitatively describe the systemic behavior of essential microbial functions is crucial for the rational design of ME applications. The study of the central carbon metabolism of species, which redirects carbon fluxes to the formation of carbon products, has become of great importance for systems biology approaches. *E. coli* is the most widely studied microbial organism, and an important species in the fields of microbiology and biotechnology, because it is used to produce useful materials in the industry and its central carbon metabolism has been studied for many years as the hub on which many catabolic and biosynthetic processes are built (Kurata et al., 2014).

The use and improvement of different mathematical techniques to describe the kinetics of the central carbon metabolism of *E. coli* has been increasing considerably over time. This can be seen as a case study to discuss the insights of this review regarding the use and evolution of dynamic models of *E. coli*, often describing its aerobic growth in continuous culture with a limiting concentration of carbon source. One of the first remarkable attempts was made by Chassagnole and coworkers, who presented the design and experimental validation of a dynamic model that deals with the lack of kinetic information on the dynamics of the metabolic reactions. This model uses experimental observations of intracellular metabolite and co-metabolite concentrations to validate the model structure and to estimate kinetic parameters (Chassagnole et al., 2002). The kinetic types and regulations of the different enzymatic reactions, with non-linear feedback/feedforward, linked for the first time the sugar transport system with the reactions of glycolysis and the pentose-phosphate pathway, using reversible Michaelis-Menten equations, Hill equation, allosteric regulation and activation, among others. Some years later, a similar approach was developed, with the integration of pathways for the tricarboxylic acid cycle and anaplerotic reactions, and including analysis of metabolic changes inside the cell in response to specific pathway gene knockouts (Kadir et al., 2010).

Although these kinetic models study and evaluate in detail many biochemical pathways, reactions and cycles, they share drawbacks, and use simplifications of complex enzymatic activities. Also, they disregard system regulatory properties, such as metabolic regulation networks, and a weak evaluation of the models by insufficient or limited types of experimental data. Peskov and collaborators proposed a more extensive and detailed model to solve these problems. They use several stages according to Cleland's classification to develop and evaluate their model (Cleland, 1963), which allowed to use *in vitro* and *in vivo* experimental data, based on fluxomics and metabolomics, to avoid the ambiguity shown in previous models caused by comparing the coincidence between predicted and experimental data (Peskov et al., 2012).

To model the kinetic behavior of the system, the reaction rate complexities depend on the catalytic mechanism, the regulatory properties allowed and the amount of experimental data available for evaluation of the predictions. Therefore, they use four levels of detail: (1) when the mode of action is simple, not metabolic regulated or when no experimental data is available to evaluate the effect of parameters (Michaelis-Menten equations); (2) when different quantitative data is available, and enzymes have complex catalytic or regulatory mechanisms, but without any allosteric properties (generalized Cleland equations); (3) when enzymes have allosteric properties (Hill equation, Monod equation, among others); and, (4) when reaction rates of enzymes cannot be expressed by a single equation, rather with a separated ODE system, considering effect of pH on enzyme activity, and using *in vitro* experimental data (Cornish and Bowden approach, Cornish-Bowden, 1979). This model is capable to suggest better hypotheses about system regulatory and functional properties, since it uses analyses of different types of experimental data. However, it takes into account a small number of reactions compared with the thousands present in a genome-scale model. A more detailed descriptions of the formalisms used can be found in (Peskov et al., 2012).

Later on, Khodayari and his team made a good effort to facilitate the construction of a larger-scale kinetic model of *E. coli*, introducing an ensemble modeling procedure to address the challenge of identifying kinetic parameter values and kinetic rate laws. The model integrates all the reactions used in previous discussed models (Chassagnole et al., 2002; Kadir et al., 2010; Peskov et al., 2012). Their method consists in decomposing metabolic reactions into elementary reaction steps and incorporating phenotypic observations (including genetic perturbations) in a parameterization scheme. The model satisfies steady-state experimental fluxomics and metabolomics data, and minimizes discrepancies between model predictions and experimental measurements. The estimation of parameters problem is solved using genetic algorithms taking into account wild-type and mutant flux data. A Michaelis-Menten equivalent formalism of the model, shows that the predicted fluxes and metabolite concentrations are within acceptable uncertainty ranges (Khodayari et al., 2014). Nevertheless, this kind of study requires the availability of additional experimental flux measurements for mutant strains having perturbations in different parts of the metabolism, in order to perform more robust parameterization of a genome-scale kinetic model. Moreover, its application is restricted to the steady state with a constant cell growth rate.

The extension and integration of new insights to previous dynamic models is a trend to improve the power of predictions. Jahan and coworkers proposed a kinetic model that uses detailed kinetic equations, with gene regulations to reproduce the dynamics of wild-type and multiple genetically modified mutants under aerobic conditions in a batch culture. At the same time, the model estimates a specific cell growth rate that is linear to the total production of adenosine triphosphate, which reflects the reconstituted metabolic pathways caused by genetic changes (and avoiding the use of Monod equation as in previous models). The values of parameters are estimated using a constrained evolutionary search method to be able to predict allosteric effectors and gene expressions. The values estimated are fixed for all the mutant cases, an improvement with respect to previous models that use different parameter values for each mutant. The dynamic model uses the structure of the batch or continuous culture based on mass balance equations in a system of ODEs, and a cell growth rate estimation connected to the flux of production of adenosine triphosphate (Jahan et al., 2016).

Another effort to include new knowledge and to improve capabilities of a dynamic model of *E. coli* was performed by Millard and his group. They developed and validated a model that links metabolism to environment and cell proliferation through intracellular metabolite levels. Also, the study explores the fact of metabolic regulation producing robust properties and a control widely distributed across the network, from a molecular level to the overall cellular physiology level. The model is based on the ones published by Kadir and Peskov, but it increases the number of pathways and the level of mechanistic detail, and also includes exchange reactions and a single reaction to model growth coupled to glucose uptake. MCA was used to validate control properties and impact of a small change in the rate of each reaction, flux and metabolite concentration (Millard et al., 2017). This study shows that deeper analyses have to be performed to ensure the validity of proposed structures.

We have seen that different mathematical models describing the same organism, do not ensure full capabilities individually. Different studies and considerations at local and global levels have to be evaluated for a dynamic model to be competitive for genome-scale applications. There has been an interest in supporting existing predictive models using approximate kinetic rate expressions and well-known structures. One example is using the lin-log approach in the kinetic model developed by Chassagnole and coworkers. This work was performed by Visser and collaborators, who compares the validity of a mechanistic model and a lin-log model derived from the mechanistic one. The study demonstrated the value of lin-log approaches as MCA extensions, since it allows to build kinetic models, based on MCA parameters, that can be used for constrained optimization problems, being valid for large changes of metabolite and enzyme levels (Visser et al., 2004; Tušek and Kurtanjek, 2010).

## Computational strain optimization (CSO)

CSO is usually seen as a bi-level framework, because its tasks are commonly divided into two stages or layers: one to perform the phenotype prediction (described in a previous section) encompassing the biological objectives of the problem, and another where the bioengineering objective is tackled in the form of an optimization problem. The objective is to find the optimal set of genetic modifications applied to an organism to achieve a desirable goal (Burgard et al., 2003).

Possible solutions need to fulfill feasibility specifications, and thus the optimization algorithm also deals with the definition of solution spaces that can be implemented *in vivo*. These CSOMs, can be based on purely constraint-based or kinetics-based modeling, or the combination of both. CSOM comprises a myriad of methods that generate possible solutions to ME tasks, namely: (1) gene deletion, by canceling fluxes related to specific genes making their reaction rates equal to zero; (2) heterologous insertion, by adding new genes or pathways, and redirecting fluxes in the desired directions; (3) gene modulation, tuning the level of contribution of enzymes by over or under expressing the activity of their reaction rates; and (4) cofactor binding specificity modulation, by exchanging the cofactor specificities of certain reactions in a network. These tasks can also be used in combination with each other to find more complex ME strategies. The solutions are translated into modifications in the network applied to the flux constraints or kinetic parameters, for constraint-based and kinetics-based models, respectively. A complete description and discussion regarding application of CSOMs for constraint-based models, with a special classification as exact bilevel mixed-integer, metaheuristic and elementary-mode analysis-based programming methods, is provided in (Maia et al., 2016).

The use of metaheuristic approaches in CSOMs brings some important advantages with respect to exact methods, such as providing a framework that can easily scale well for bigger models and larger numbers of modifications, which is computationally less costly and possibly faster for finding sufficiently good solutions. Another remarkable feature is to gain a level of flexibility to implement complex frameworks, with an independent implementation of phenotype prediction and strain optimization layers, which can allow, for example, to implement nonlinear, or even discontinuous, objective functions in the optimization layer to define more significant problems. Common approaches combine the assumption that microorganisms naturally maximize their growth, with simulated annealing or EAs to select genetic manipulations that will result in a desirable high productivity goal (Rocha et al., 2008).

### CSO using dynamic models

For industrial biotechnology purposes, a mathematical model must be able to simulate, predict and examine a variety of scenarios where a biological system is operating under certain assumptions and environmental conditions. It is possible to design CSOMs based on dynamic models through the study of the transient and equilibrium states of the system. The main goal of these CSOMs is to find forms of reaction rates, i.e. parameter values for defined kinetic expressions, for which a system has the best fitness with respect to a certain phenotype, e.g., an improvement in the production of a particular compound.

*In silico* ME strategies are designs that represent a way of improving the performance of an organism toward a specified goal. They can include the use of dynamic models that predict the behavior of the system under the influence of perturbations, such as gene deletions, enzyme modulations or changes in the medium conditions. The selection of a dynamic model with high power of prediction benefits the design of newly engineered microbial strains. CSO using dynamic models aims at identifying proper gene deletions or levels of enzymatic activity applied to microbial processes.

The methods can be based on two types of formulations, exact or stochastic. On the side of exact formulations, a basic approach involves linear programming problems with linear objectives and constraints defined in a convex space. However, most of the optimization problems applied to biological systems introduce non-linear programming problems over continuous or discrete variables. This means that the search process can take place in non-convex spaces, resulting in the possible existence of multimodality, i.e., the existence of multiple local solutions. This type of problems belongs to the class of non-deterministic polynomial-time hard problems, which are computationally more complex and less efficient to solve than polynomial-time ones (Erickson, 2009).

Exact methods are always able to yield the optimal solutions, but their computational time increases exponentially with the size of networks and of the solutions, thus, demanding the development of approximate and faster algorithms. These include exact Mixed-Integer Linear Programming formulations, that can be combined with approximation methods such as generalized linearization of kinetic models (Vital-Lopez et al., 2006). Further, global optimization of non-linear dynamic models has been explored by recasting the system into an equivalent generalized mass action model, which facilitates the numerical computation for the optimization task to identify genetic modifications (Pozo et al., 2011).

On the other hand, stochastic global optimization can be used to locate solutions near to the global optimum, including EAs which have shown acceptable performance for applications in biological systems (Banga, 2008; Rocha et al., 2008). Examples of stochastic optimization include: applications of robust methods for parameter estimation in nonlinear dynamic systems, that outperform significantly methods previously used for three specific benchmark problems (Rodriguez-Fernandez et al., 2006); EAs for predicting optimal reaction knockouts and enzyme modulation strategies for the maximization of serine production by *E. coli* (Evangelista et al., 2013); and, the exploration of a computational environment where dynamical models are used to support simulation and optimization tasks, by using metaheuristics to identify modifications of parameters so that the production of dihydroxyacetone phosphate is maximized in *E. coli* (Evangelista et al., 2009). Usual alternatives to characterize targets include making a local parameter sensitivity analysis, or simulating more significant changes in enzyme levels or other elements.

Moreover, novel algorithms use multi-objective dynamic optimization to identify combinations of targets (enzymatic modifications) and the degree of modulation to optimize a set of pre-defined performance metrics, subject to process constraints (Villaverde et al., 2016b). These methods were demonstrated on a realistic metabolic model of chinese hamster ovary (CHO) cells, used for antibody production, while sustaining a robust growth in CHO cells, increasing biomass production, product titer, and keeping the concentrations of lactate and ammonia at low levels. Additionally, exhaustive studies of dynamic models have been developed, such as a kinetic model of CHO cell metabolism (Nolan and Lee, 2011), together with a novel framework for simulating the dynamics of metabolic and biosynthetic pathways of these cells grown in fed-batch culture. The authors later complemented their study with a method to simultaneously identify processes and cell modifications that improve antibody production, by exploring combinations of process variables and knock-outs applied to the CHO model (Nolan and Lee, 2012).

Table 2 summarizes examples of applications of CSOMs using dynamic modeling, using the exact or stochastic methods mentioned above. The type of formulation includes the phenotype prediction method and programming approach used to solve the optimization problem. From a comparative perspective, we will analyze two examples of strain optimization that have the same objective, the maximization of serine production in *E. coli* model (Chassagnole et al., 2002), but that use a different formulation to find a set of modifications to reach such goal. One uses a linear approximate version of the non-linear model and gets an exact solution for the overproduction problem, and the other one uses the non-linear model and a metaheuristic approach to solve the strain optimization part.

**Table 2:** Applications of computational strain optimization methods using dynamic models.

**Description**	**Type of formulation**	**Case studies**	**Evaluation**	**References**
Linearization of kinetic model and iterative optimization	Approximate, mixed-integer linear programming	Serine overproduction in *E. coli*	Theoretical (experimental evidence)	Vital-Lopez et al., 2006
Metaheuristics to identify modifications of parameters	Stochastic, evolutionary computation	Maximize production of dihydroxyacetone phosphate in *E. coli*	Theoretical (experimental evidence)	Evangelista et al., 2009
Brute-force to simultaneously identify process and cell modifications	Exact, exhaustive search	Improvement of antibody production in Chinese Hamster Ovary cells	Experimental	Nolan and Lee, 2012
Metaheuristics to identify modifications of parameters	Stochastic, evolutionary computation	Maximization of serine production by *E. coli*	Theoretical (experimental evidence)	Evangelista et al., 2013
Multi-objective dynamic optimization	Exact, mixed-integer nonlinear programming	Sustained and robust growth of Chinese Hamster Ovary cells	Theoretical (experimental evidence)	Villaverde et al., 2016b

Vital-Lopez and collaborators used dynamic modeling of metabolism to find optimal engineering interventions. The procedure relies on the generalized linearization of a dynamic model, by employing a Lagrange expansion, and the iterative application of mixed-integer linear programming optimization to hierarchically identify reaction eliminations and/or enzyme level modulations (Vital-Lopez et al., 2006). The authors find that the resulting engineering strategies and robustness depends mostly on the range of bounds defined for the metabolite and enzyme levels. Narrow bounds on concentrations generate accurate predictions, while for larger concentration ranges there was substantial divergence between the non-linear and the linearized model predictions. This means that local information is not sufficient to identify optimal manipulations when wider changes in enzyme levels are allowed. The strategy permits to find strategies with good results, since the flux product was increased in some cases for more than twice its value with respect to the wild-type. The linearization approach helps to simplify the optimization problem, and its exact formulation ensures that the solution is optimal. This procedure is a tool that can be used for any dynamic model.

In an alternative approach, Evangelista and co-workers used EAs for predicting optimal reaction knockouts and enzyme modulation strategies for the same case study. The algorithm used a non-linear model to reach a set of solutions with higher quality, as compared to the one described above, concluding that, as the number of reaction modification increases, the marginal product flux gain usually tends to decrease (Evangelista et al., 2013). The solutions are not constrained by flux or concentration bounds since the non-linear model ODEs are simply integrated, assuming that the model depicts adequately the subjacent reality. This approach does not ensure finding the optimal solution, but improves the quality of solutions in most cases, with respect to the linearization approach.

The evaluation of results from strain optimization methods with respect to experimental data is a key process to determine how effective an approach based on dynamic models is. The methods described in Table 2 contain strain optimization procedures that were mostly demonstrated theoretically, despite some experimental evidence being presented for all of them. In the work by Nolan and Lee (2012) involving CHO cells and antibody production, the authors produced an experimental evaluation, confirming that changes in parameters are supported by experimental results on the modulation of lactate dehydrogenase activity. They show that by reducing the enzyme activity, lactate production decreases, and cell density and antibody production increase (>2-fold titer improvement), in good agreement with experimental data. The authors conclude that the benefits of cell engineering (enzyme activity modulations) directly depend on the process parameters, and that dynamic metabolic model is necessary to efficiently explore design spaces for cell and process modifications.

## Hybrid models

During the last decades, there has been an interest in combining the advantages of distinct types of modeling to improve phenotype prediction, using hybrid models. These hybrid approaches suggest promising ways to tackle limitations from dynamic and constraint-based models, merging capabilities and exploiting knowledge of already well-known processes, parameters and constraints, and to decrease costs, improve efficiency and reach desirable phenotypes. It is expected to have future results using methods focused on improving limitations of the most promising approaches, especially the ones incorporating kinetic models and detailed information (i.e., network structures, kinetic rate expressions and parameter values). These quantitative methods are able to describe, predict and optimize the behavior of a biological system, in comparison with the approach that CBM offers for pathway-oriented analysis that involves only prediction of the steady state fluxes.

Different researchers have developed hybrid models joining stoichiometric information with good-quality kinetic data. Table 3 shows examples of using hybrid models only for phenotype prediction or for strain optimization; the type of formulation includes the programming approach used to solve the optimization problem, and whether they were not only theoretically, but also experimentally evaluated. These methods were originally proposed by Covert and collaborators to improve FBA predictions, in the case of having regulatory effects with a dominant influence, for which transcriptional regulatory discrete events are included as time-dependent restrictions to constraint-based models, and applied to the illustration of systemic effects such as catabolite repression, the aerobic/anaerobic diauxic shift and amino acid biosynthesis pathway repression (Covert et al., 2001). Later on, a complete hybridization was made by Mahadevan and coworkers, describing the dynamic behavior of a metabolic system using an extension of FBA, namely dynamic FBA (DFBA). This approach was focused on the analysis of diauxic growth in *E. coli*, by reprogramming the metabolic network and studying the transience of metabolism (Mahadevan et al., 2002). DFBA takes into account dynamic information, and can provide useful insights for the design of cell factories in ME applications. However, the optimization problem formulated as a non-linear programming problem results in low scalability, since, as the size of the network increases, the number of variables also increases leading to a problem with much higher complexity to be solved. Moreover, thermodynamics have been added to constraint-based models by Beard and collaborators, who improved the power of prediction of stoichiometric models for large-scale metabolic networks, coupling steady-state information with dynamic equations of thermodynamic constraints, such as energy conservation, enthalpy, entropy and Gibbs free energy. Classical concepts in equilibrium thermodynamics are generalized to non-equilibrium settings (Beard et al., 2002; Qian and Beard, 2005).

**Table 3:** Examples of hybrid models used only for phenotype prediction or for computational strain optimization.

**Description**	**Type of formulation**	**Task**	**Case studies**	**Availability**	**Evaluation**	**References**
Dynamic Flux Balance Analysis (DFBA)	Exact, nonlinear programming	Phenotype prediction	Diauxic growth in *E. coli*	The COBRA Toolbox^1^ implemented in MATLAB (Schellenberger et al., 2011)	Experimental (Hanly et al., 2013; Flassig et al., 2016)	Mahadevan et al., 2002
Integrated DFBA	Exact, linear programming	Phenotype prediction	Integrated signaling, metabolism and transcription regulation in *S. cerevisiae*	Not available	Theoretical (experimental evidence)	Lee et al., 2008
Integrated Flux Balance Analysis	Exact, linear programming	Phenotype prediction	Metabolism, regulation and signaling of *E. coli* model	SimTK^2^ implemented in MATLAB	Theoretical (experimental evidence)	Covert et al., 2008
Integration of kinetic expressions as constraints into DFBA	Exact, nonlinear programming	Strain optimization	Production of glycerol and ethanol in *E. coli*	Not available	Theoretical (experimental evidence)	Gadkar et al., 2005
Dynamic strain scanning optimization for balanced yield, titer and productivity	Exact, nonlinear programming	Strain optimization	Production of succinate and 1,4-butanediol in *E. coli*	Framed GitHub repository^3^ (implementation in Python)	Theoretical (experimental evidence)	Zhuang et al., 2013
k-OptForce: integration of kinetics with Flux Balance Analysis	Exact, mixed-integer nonlinear programming	Strain optimization	Production of L-serine in mutant *E.coli* and triacetic acid lactone in mutant *S. cerevisiae*	Not available	Experimental Khodayari et al., 2015	Chowdhury et al., 2014

1 The COBRA Toolbox. “Dynamic FBA”. Opencobra.github.io. https://opencobra.github.io/cobratoolbox/stable/modules/analysis/dynamicFBA/index.html?highlight=dynamicfba

2 SimTK. “Integrated Flux Balance Analysis Model of Escherichia coli”. SimTK.org. https://www.simtk.org/projects/ifba/

3 *Framed GitHub repository. “A python FRAmework for Metabolic Engineering and Design”. Github.com. https://github.com/cdanielmachado/framed*.

Moreover, Lee and collaborators propose an integrated DFBA strategy which requires an integrated stoichiometric reconstruction of signaling, metabolic and regulatory process in *S. cerevisiae*, and incorporating kinetic parameters based on typical time scales observed in literature. The method quantitatively analyzes systematic effects of extracellular cues on cellular phenotypes and generates comparable time-course predictions when contrasted with an equivalent kinetic model (Lee et al., 2008). In addition, Covert and co-workers combined FBA with regulatory Boolean logic, and ODEs to create an integrated model of *E.coli* to describe in detail carbohydrate uptake control and behavior of diauxic growth. This approach was able to give more accurate phenotypes than a purely ODE-based model (Covert et al., 2008).

In addition, DFBA method has not only been confirmed theoretically, but also evaluated experimentally, explained in the two following examples. One example of this is its use for optimization of yeast models using a parallel bioreactor system to determine the optimal aerobic and anaerobic switching time and level of oxygen for maximal ethanol production in batch culture, and comparing results after experimental procedures. The conclusions of this study shows that parallel fermentation is a powerful tool for batch culture optimization when used in conjunction with dynamic metabolic models, where a batch culture bioreactor can be scaled-up to 5-fold change (Hanly et al., 2013). The second example is DFBA modeling formulation for accumulation of high-value storage molecules in microalgae that provides quantitative predictions under various light and nutrients. The accuracy of predictions is evaluated through independent experimental data followed by a fed-batch optimization, showing an increase of biomass and β-carotene density by factors of about 2.5 and 2.1, respectively (Flassig et al., 2016).

### CSO using hybrid models

Hybrid models can be incorporated into CSOMs to improve ME tasks, since kinetic information is used to perform more descriptive phenotype predictions as discussed before. Some efforts that have used this hybrid approach start with the work by Gadkar and his group, who added kinetic expressions as constraints into DFBA to optimize the concentration of a certain product yield (maximization of glycerol and ethanol concentrations in *E. coli* model). This was possible by analyzing an optimal temporal flux profile of a manipulated reaction. They also examined growth inhibition due to variations in the end production, concluding that genetic alterations are critical from the standpoint of productivity (Gadkar et al., 2005). Another contribution was made by Zhuang and collaborators, who built a dynamic strain scanning optimization for designing microbial strains with balanced yield, titer and productivity, applied to a study case for the maximization of succinate and 1,4-butanediol in *E. coli*. This method uses DFBA to evaluate the relationship between growth yield, growth rate, product yield, volumetric productivity, titer and economic viability of designed strains (Zhuang et al., 2013).

One outstanding effort to build CSO using hybrid models is k-OptForce (Chowdhury et al., 2014), a hybrid approach for the integration of kinetics with FBA for strain design, developed recently to increase the accuracy of predictions and conveniently using kinetic rate expressions. This framework redistributes fluxes in the metabolic network instead of aiming at an optimal value of an objective function, in contrast with pure CBM methods. The method segments the reactions of the studied metabolic network in two sets: (1) ϕ^*stoic*^, information, restricted by mass balance and thermodynamics, and (2) ϕ^*kin*^, that contains the reactions for which kinetic information is known, such as enzyme activity, kinetic parameters and metabolite concentrations, representing them as a system of nonlinear ODEs.

Afterwards, the problem to be solved with k-OptForce is formulated in two steps: (1) the wild-type behavior is defined by using flux variability analysis (Mahadevan and Schilling, 2003) for ϕ^*stoic*^, and by solving the system of ODEs to get a flux distribution at steady-state for ϕ^*kin*^. Additionally, the overproducing strain is defined under the available restrictions for concentrations and kinetics; (2) the problem is formulated similarly to the one described by the predecessor CSOM OptForce (Ranganathan et al., 2010), by transforming it into a mixed-integer nonlinear optimization problem. Taking into account the kinetic constraints, the solution comes from the computation of MUST and FORCE sets, i.e. the set of reactions required to achieve a user-defined production yield and the minimal set of reactions that need to be forced via genetic manipulation, respectively. k-OptForce was theoretically evaluated in comparison to OptForce, an exact CSOM based on pure constraint-based models, for the prediction of L-serine in mutant *E. coli* and triacetic acid lactone in mutant *S. cerevisiae*. The study revealed that the non-intuitive modifications identified for key enzymes by k-OptForce, produce less rearrangements of the flux distribution toward the product of interest, which will not exceed concentration bounds and cannot be captured by stoichiometry-alone analysis. The method is versatile enough to incorporate available omics information to further improve prediction fidelity. However, sensitivity analysis has to be performed since it is shown that the required number of interventions can be significantly affected by changing the imposed bounds on metabolite concentrations (Chowdhury et al., 2014).

Furthermore, among the methods for strain optimization presented in Table 3, which were theoretically evaluated, the developers of k-OptForce also discuss a case study that is evaluated with experimental data. Khodayari and his team use a hybrid model of *E. coli* to study succinate overproduction through strain design. The method identifies minimal interventions that improve succinate yield under both aerobic and anaerobic conditions to test the fidelity of model predictions under both genetic and environmental perturbations. Under aerobic condition, this method identifies interventions that match existing experimental strategies, finding unexplored flux re-directions such as routing glyoxylate flux through the glycerate metabolism to improve succinate yield. These interventions are able to be pointed by using kinetic descriptions that would not be discoverable by a purely stoichiometric formulation. However, under anaerobic condition, k-OptForce cannot identify key interventions because the pathways were not properly parameterized as only aerobic flux data were used in the model construction. In conclusion, this study reveals the importance of condition-specific model parameterization and provides insight on how to use kinetic models to correctly analyze the response to multiple environmental perturbations. In general, however, the number of intervention strategies when implementing hybrid approaches is a trade-off for improving computational performance (Khodayari et al., 2015).

## Discussion

In this work, we have explored the importance of developing detailed dynamic models able to support accurate phenotype predictions and their use in efficient strain optimization algorithms, a field that has the potential to produce significant impact in industrial biotechnology. Methods to fulfill these tasks and to generate new knowledge are emerging and being evaluated, with the intention of exploring and bridging the gap between different (bio)mathematical modeling frameworks. Systems biology provides strategies for ME to take advantage of the best simulation and optimization method features, and to deal with the most remarkable limitation regarding the lack of available experimental information, which affects accuracy and feasibility of solutions (Machado et al., 2012). Another identified issue is the addition of more detailed information to already existing genome-scale models, to increase their scalability and the range of industrial applications for strain design. The global challenge consists in generating high quality models that make a difference in the improvement of performance of CSOMs for larger scales. However, this task becomes a challenge since metabolic simulation has to deal with thousands of reaction rates and metabolite concentrations.

Moreover, current research trends point to the inclusion of parameter uncertainty to increase the level of flexibility, using kinetic models built with stochastic optimization methods, and also improvement of phenotype predictions by using complementary data, such as various types of omics data, particularly gene expressions (Jahan et al., 2016). Furthermore, some methodologies include evaluation of stability, robustness and other type of analyses of dynamic features, such as oscillations (Schaefer et al., 1999). Additionally, identifiability analysis helps to drive dynamic models to the development of better experimental techniques and to polish methods to solve the optimization problems, focusing on exact or stochastic formulations (Villaverde and Barreiro, 2016).

The revised dynamic modeling approaches are supported by the use of optimization methods for two main identified tasks. On one hand, we have the development of models for phenotype prediction, particularly for parameter estimation. This task requires model calibration through the minimization of differences between predicted and experimental values (Banga, 2008). On the other hand, we have the strain design task, which aims at finding the optimal interventions strategies for producing strains with enhanced capabilities (Vital-Lopez et al., 2006). Both tasks have to deal with choosing the most suitable optimization method, which depends on the type of problem or application. However, it has been noticed that stochastic optimization approaches seem to be an acceptable option to avoid issues with scalability, flexibility or convergence time. Involving metaheuristics, the search of alternatives in bigger solution spaces is more efficient for complex (multi)objective functions. For example, finding the optimal kinetic parameters to reach desired phenotypes in a genome scale, once a suitable known model structure is identified, such as central carbon metabolism of *E. coli* or *S. cerevisiae* (Rocha et al., 2008).

Kinetic models can also be studied within hybrid models for phenotype prediction, which allows the integration of available kinetic relations with genome-scale constraint-based model formalisms. This idea results in more detailed descriptions of large-scale models, since the effect of metabolite concentrations and substrate-level enzyme regulation cannot be captured with stoichiometry-only metabolic models and analysis methods (Chowdhury et al., 2014). These models can be used as a base for optimization methods for strain design to identify genetic perturbations that are consistent with enzyme expressions and metabolite concentrations. In principle, the algorithms can perform the combination of CSOMs that use only constraint-based or only kinetics-based models. The integration of approaches has become very promising to accelerate the process of innovation in the world of ME, leading to the targeted overproduction of desired chemicals.

The mentioned CSOMs using hybrid models, such as the one used by Chowdhury and coworkers, attempt to identify a minimal set of interventions on enzymatic parameter changes and reaction flux changes, such that less rearrangements of the flux distribution are required, and concentration bounds are not violated (Chowdhury et al., 2014). An important remark is that CSOMs with hybrid approaches can always be improved incorporating available omics information, to sharpen the prediction fidelity. More constraints can be added to the optimization problems to restrict more the flux ranges and to decrease the space of possible solutions. Also, temporal consideration can be addressed by integrating hybrid CSOMs with the DFBA framework (Mahadevan et al., 2002) to explore the variation of metabolic modifications as a function of time suggesting similar actions of ribonucleic acid interference type of interventions.

We emphasize as one of the major outcomes from the revision of phenotype prediction and strain optimization methods, the fact that the selection of a specific dynamic modeling approach is always subject to the prospective application, as well as the amount and type of experimental data available. While and adequate path is difficult to define for all cases, Table 1 can provide an aid in this process. For instance, mechanistic dynamic modeling has been widely used to have conventional expressions describing the structural features of metabolic systems. However, this represents a general approach that might not be able to detail specific biological processes, for which other methods can be used. One example of this is the use of log-linear approximations to accurately describe dynamic responses of poorly known non-linear systems, however, parameter estimation methods might be limited in their ability to satisfactorily fit data from observations when small concentration of metabolites are present. This kind of problem can be tackled using convenience rate laws, since they require a small number of parameters that can be easily computed. Additionally, cooperativity and saturation expressions are used to fit experimental data for systems with a saturable form, while modular rate laws can simplify thermodynamic-kinetic modeling formalisms. By providing analytical solutions and avoiding the use of non-linear problems, the computational burden and convergence times are greatly reduced.

Furthermore, stochastic approaches are able to capture variability in the described species, for very well-known structural features, such as stiff systems. The dynamics are simulated by knowing the probabilities of transitions between every possible state. However, the formulation can become a non-trivial stochastic simulation algorithm for networks when the number of reacting molecules is large per reactant, which is common for most of realistic kinetic models. The aim will be always to establish a trade-off between the size of the model and the level of accuracy of the solutions, depending on the type of experimental data, which is not always available in the literature. Finally, efforts for enforcing the inclusion of experimental data as Supplementary Material when publishing new findings should be taken by publishers, a measure that would greatly improve the development of these type of methods.

Also, studying kinetic models, especially used within hybrid models, has revealed strengths and limitations of model-driven strain design, and indicated that kinetic models have the potential to substantially over-perform FBA-based predictions when parameterized under similar conditions, but may perform worse than FBA when predicting a significantly different metabolic phenotype. Studies have also demonstrated the need to perform model parameterization for a diverse range of genetic or environmental perturbations, and the tight integration of transcriptional level along with substrate-level regulatory interactions. At a fundamental level, kinetic models must be *a priori* provided with the quantitative description and as many as possible regulatory switches in response to genetic or environmental perturbations. The quality of mechanistic information enables a detailed description of metabolism such as dynamics, enzyme activities, and metabolite concentrations but can result in erroneous predictions since some modeling assumptions can be missing or incorrect. Nevertheless, by studying failure modes of kinetic models, valuable information can be uncovered for restoring prediction consistency for new phenotypes (Khodayari et al., 2015). These findings, together with reported experimental evaluations of constraint-based CSOMs, available in the literature since the last two decades (Maia et al., 2016), help building the case for the combination of these approaches with kinetics-based models, becoming tools for the optimization of bioprocesses for a wide range of industrially relevant chemicals.

Additional studies, not covered in detail in this review, point to the use of artificial intelligence as a different approach to analyze mathematical models for ME purposes. Novel technologies applied to metabolomics can substantially improve search algorithms to increase the dynamic range, number of carbon-carrying metabolites and possible pathways to transform a given source metabolite into a given target metabolite (Kell, 2006). One example of the use of artificial intelligence to accelerate the design of microbial cell factories, is the development of an efficient workflow for combinatorial optimization of the large biosynthetic genotypic space of heterologous metabolic pathways in yeast. This method is able to precisely tune the expression level of genes with a machine learning algorithm based on an artificial neural network ensemble to avoid over-fitting, and it is also able to predict strains with titer improvements among several possible designs (Zhou et al., 2018). Another recent advance on exploiting artificial intelligence techniques is an approach that combines machine learning and abundant multiomics data (proteomics and metabolomics) to effectively predict pathway dynamics. The method outperforms a classical Michaelis–Menten kinetic model, and produces qualitative and quantitative predictions that can be used to productively guide bioengineering efforts, by using only two time-series as training data. This work shows that, given sufficient data, the dynamics of complex coupled non-linear systems can be systematically learned (Costello and Martin, 2018). Finally, the development of biological models based on artificial intelligence has been analyzed in a recent review, which highlights the scope of information collections, database constructions, and machine learning techniques that can facilitate strain design (Oyetunde et al., 2018).

In this review, we analyzed the main mathematical formalisms used for dynamic/hybrid modeling of microbial metabolism, scrutinized their inclusion into strain optimization applications and made a critical evaluation of future steps in this research topic. The ladder toward more realistic strain engineering strategies seems to be undeniably limited by more detailed representation of the dynamics of the biological systems. However, as was thoroughly discussed in this work, these are still hindered by the lack of appropriate parameters even for the most studied organisms such as *E. coli* and *S. cerevisiae*. Thus, the advancement of the field will always be dependent on incremental investments in fundamental research geared toward the deeper understanding of biological mechanisms, including local phenomena. For this purpose, the use of hybrid models as flexible vessels for the cumulative inclusion of knowledge will be of major importance in the coming years.

## Author contributions

All authors participated in the preparation of this work. OK had a major role in writing and editing the manuscript. All authors contributed to manuscript revision, read and approved the submitted version.

### Conflict of interest statement

The authors declare that the research was conducted in the absence of any commercial or financial relationships that could be construed as a potential conflict of interest.

## Abbreviations

CBM: constraint-based model
CHO: chinese hamster ovary
CSO: computational strain optimization
CSOM: computational strain optimization method
DFBA: dynamic flux balance analysis
EA: evolutionary algorithm
EC: elasticity coefficient
FBA: flux balance analysis
FCC: flux control coefficient
MCA: metabolic control analysis
ME: metabolic engineering
ODE: ordinary differential equation.
